# Active Confirmation Bias in the Evaluative Processing of Food Images

**DOI:** 10.1038/s41598-018-35179-9

**Published:** 2018-11-15

**Authors:** Kajornvut Ounjai, Shunsuke Kobayashi, Muneyoshi Takahashi, Tetsuya Matsuda, Johan Lauwereyns

**Affiliations:** 1 Graduate School of Systems Life Sciences, Kyushu University, Fukuoka, 819-0395 Japan; 2 Department of Neurology, Fukushima Medical University, Fukushima, 960-1295 Japan; 3 Tamagawa University Brain Science Institute, Tokyo, 194-8610 Japan; 4 Faculty of Arts and Science, Kyushu University, Fukuoka, 819-0395 Japan

## Abstract

Predictive processing is fundamental to many aspects of the human mind, including perception and decision-making. It remains to be elucidated, however, in which way predictive information impacts on evaluative processing, particularly in tasks that employ bivalent stimulus sets. Various accounts, including framing, proactive interference, and cognitive control, appear to imply contradictory proposals on the relation between prediction and preference formation. To disambiguate whether predictive cues produce congruent biases versus opponent mechanisms in evaluative processing, we conducted two experiments in which participants were asked to rate individual food images. The image database included appetitive and aversive items. In each trial, a cue predicted, with varying degrees of reliability, the valence of the impending food image. In both experiments, we found that the ratings exhibited congruent biases as a function of the reliability of the predictive cue, with the highest evaluations following the most reliable positive-valence predictions. Eye prepositioning further showed a selective spatial bias suggestive of response preparation in line with the predictions. The response times also exhibited a pattern of results consistent with selective preparation, producing slow responses following invalid predictions. The data suggested an active form of evaluative processing, implementing a confirmation bias that aims to accommodate the prediction.

## Introduction

In contemporary psychology, neuroscience, and philosophy of mind, the most comprehensive accounts of brain and mind suggest that predictive processing is central to many, if not all, forms of cognition^1–5^. Also with respect to evaluative processing, it has been shown that contextual information can elicit expectations that modulate the liking of stimuli as diverse as visual art^6^, music^7^, coffee^8^, soda^9^, and wine^10^. The effects of prior expectations appear to have a fundamental impact on the perceptual processes underlying the evaluation, in ways that are difficult to accommodate with normative theories about rational decision-making^11^.

Instead of rational decision-making, evaluative processing often involves complex or seemingly inscrutable processes we usually call “subjective,” with the implication that they are idiosyncratic and cannot be disputed (as according to the Latin maxim, *De gustibus non est disputandum*; “In matters of taste, there can be no disputes”). However, in reality we frequently do debate evaluative processing in virtually all aspects of human society, from commerce to health and well-being, from bioethics to politics. The phenomena of evaluative processing warrant scientific investigation, particularly with a view to understanding how, and when, biases distort the evaluation of objects and events that afford no easy objective metric^5,12–14^. The issue is to chart the extent to which evaluative processing is pervious to external influences. The present study aims to contribute to this task.

More specifically, we focus on the direction of influences from prior expectations when both the predictions and the outcomes are bivalent. We chose food images as a relevant category for evaluative processing with bivalent items^15–17^ that elicit a complex integration of visual and non-visual features (e.g., flavor, caloric value, nutritive attributes)^18–21^. Previous research in our lab had further shown that the rating of individual food items provides a suitable opportunity to investigate the cognitive mechanisms underlying evaluation^22^. Here, we examine how explicit, supraliminal positive or negative predictions impact on the evaluation of appetitive or aversive food images.

Previous research on framing effects might be taken to suggest that bivalent predictions should lead to congruent biases in the evaluative processing, with more positive evaluations following positive predictions and vice versa^23–27^. We term this the “congruent bias” hypothesis. The congruent biases from framing would be due to explicit expectations under voluntary control. It has further been suggested that such positive or negative expectations evolve over time, potentially becoming more polarized^28,29^. Other phenomena and theories can also be interpreted to imply similar congruent biases, albeit on the basis of implicit mechanisms that would be activated automatically or unconsciously by the predictive cues. These phenomena and theories include priming^30–32^, proactive interference^33^, Pavlovian-instrumental transfer^34,35^, and emotional contagion^36,37^.

In contrast, predictive cues could lead to the activation of opponent mechanisms, aiming to operate explicitly against external influences. We term this the “opponent mechanism” hypothesis. For instance, proactive control would function as an endogenously activated mechanism in opposition to biasing information^38,39^. Previous research has identified a number of conditions in which the evaluative processing counteracted the biasing influences^7,40–42^. Here, the opponent mechanism hypothesis implies a deliberate effort to inhibit responses in line with the prediction. Such active inhibition, however, may produce opposite effects, promoting responses that run counter to the cueing, analogous to phenomena such as inhibition of return^43^ and negative priming^44^. Thus, the evaluation of appetitive food images would be higher following negative predictions than following positive predictions. Conversely, aversive food images, when occurring in contrast to the prediction, would be evaluated more negatively than aversive food images that appear in agreement with the prediction.

Counteractive evaluative processing might also occur as a function of more implicit opponent mechanisms, consistent with the notion of prediction errors in the neuroscience literature on dopaminergic mechanisms (e.g., an unexpected reward leads to stronger dopaminergic activation than an expected reward; such activation is thought to reflect learning and adaptive evaluative processing)^45–48^. Here, the conjecture would be that the predictive context amplifies the evaluative processing of unexpected outcomes.

## Rationale of the Present Study

**Figure 1:**
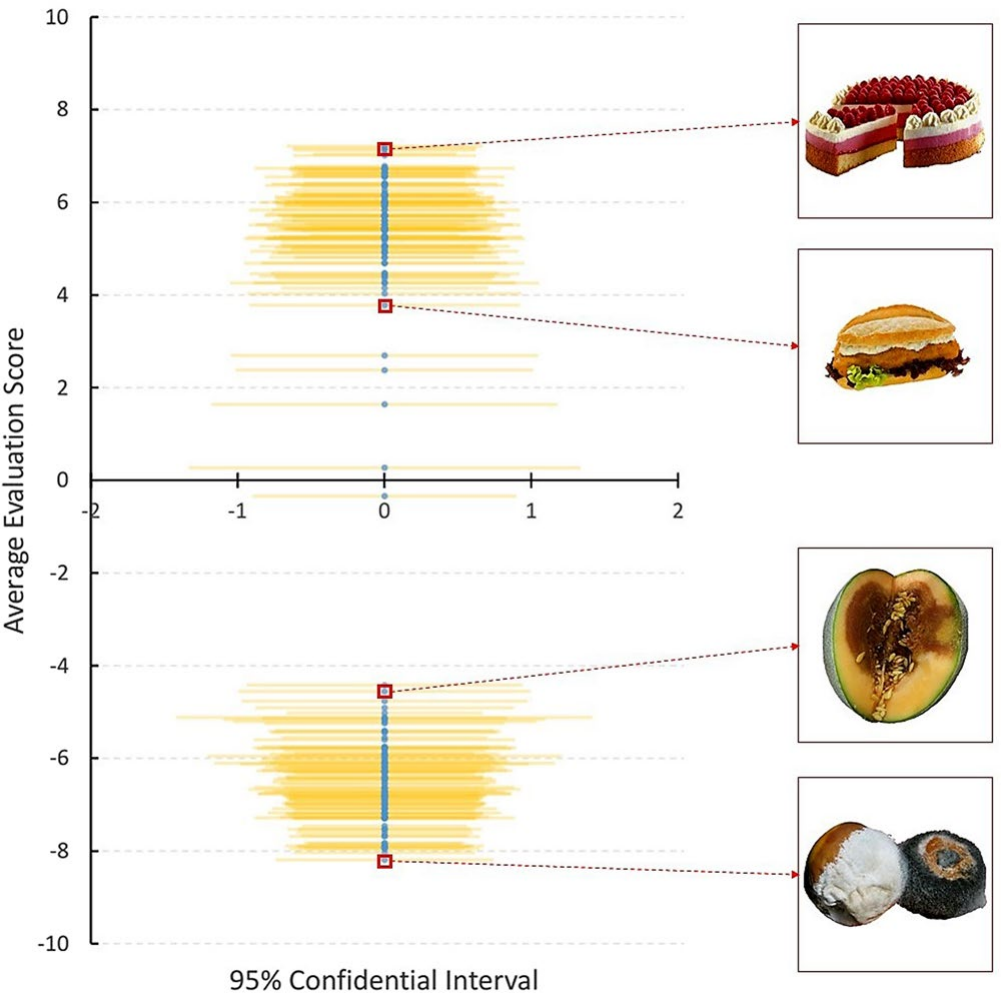
Distribution of the bivalent stimulus set. The blue points and yellow lines reflect the average evaluation scores (blue, vertical axis) and the associated 95% confidence intervals (yellow, horizontal axis) of the 200 images in the database, collapsed across all conditions and all subjects in the present study. Four representative images are shown in inset figures on the right.

**Figure 2:**
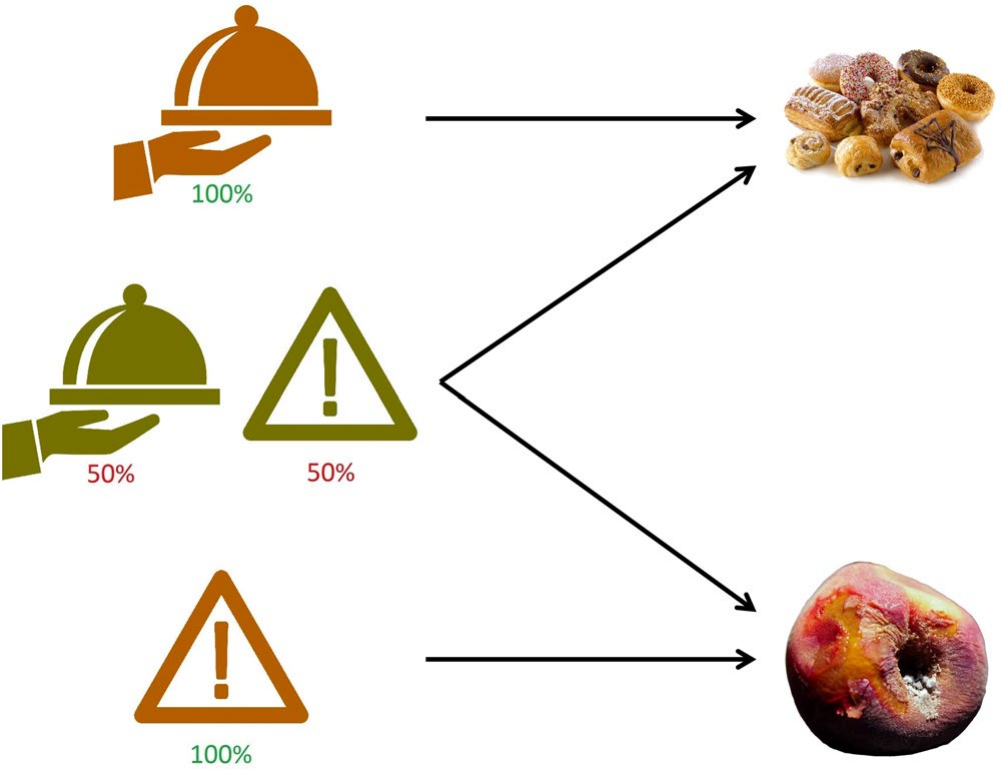
Schematic representation of the predictive cues in Experiment 1, associated with different outcome types. The color indicates the level of reliability (orange, 100%; green, 50%) whereas the icon indicates the valence (tray, positive; hazard, negative). For 50% reliable cues, the predicted valence reflects merely framing.

**Figure 3:**
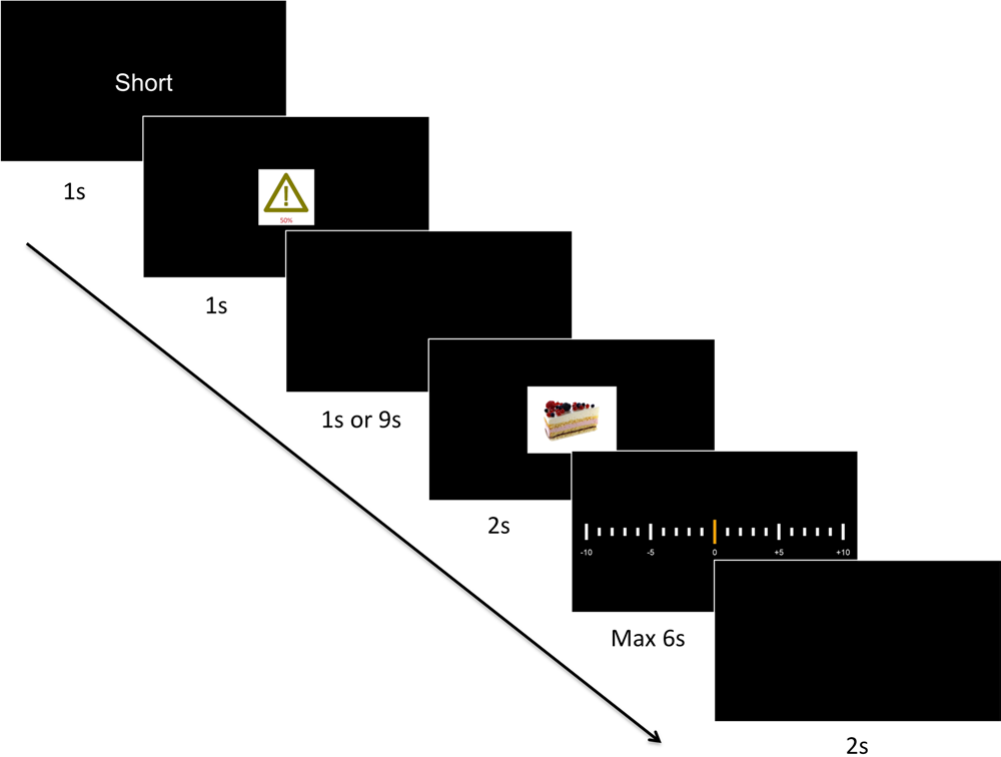
Trial structure in Experiment 1. Each trial starts with a word cue, presented for 1 s, indicating whether it will be either a short- or long-delay trial. The predictive cue is shown for 1 s, followed by a blank screen for either 1 s (short delay) or 9 s (long delay). The target image is shown for 2 s at the center of the screen, followed by the evaluation bar. Participants have maximally 6 s to respond by bending the joystick. The response is followed by a 2 s inter-trial interval.

The present study was explicitly designed to pitch the congruent bias hypothesis against the opponent mechanism hypothesis. We conducted two experiments in which subjects received a predictive visual cue in advance of a single food image in each trial. The subjects were asked to rate the food image on a scale from −10, for most aversive, to +10, for most appetitive. The database of food images included a balanced variety of images to cover the entire range on the bipolar scale (see Fig. 1). The predictive cue indicated, with varying degrees of reliability, whether the impending food image would be aversive or appetitive. In Experiment 1, the predictive cue was either perfectly reliable or random (see Fig. 2). Thus, in Experiment 1, random cues effectively offered only a positive or negative framing. After the presentation of the food image, the subjects could manipulate a joystick to record their evaluation (see Fig. 3). In Experiment 2, the predictive cue was either 75% reliable or explicitly neutral. In both experiments we recorded manual responses and eye positions.

According to the congruent bias hypothesis as a function of framing, the evaluations for appetitive items would be the highest for perfectly reliable predictions, and higher following positive framing than following negative framing. For aversive items, the evaluations would be the lowest for perfectly reliable predictions, and lower following negative framing than following positive framing. Given that framing would depend on voluntary control, the size of the framing effects should depend on the reliability of the predictive cues. Framing is also hypothesized to lead to response preparation (i.e., anticipatory eye positioning toward the expected response side), and likewise influence response time, with faster responses when the valence of the target matches with the prediction than when there is a mismatch between prediction and outcome. Theories and phenomena that predict congruent biases on the basis of implicit mechanisms do not predict any influence of the cue’s predictive validity, nor do they predict any active response preparation (i.e., no prediction of effects on response time or eye prepositioning).

According to the opponent mechanism hypothesis, the evaluations for appetitive items would be higher following negative framing than following positive framing, and again higher following positive framing than following perfectly reliable predictions. That is, when unexpected or running against the prediction, a positive item would elicit the largest counteractive amplification and therefore receive the most favorable evaluation. Analogously, the evaluations for aversive items would be lower following positive framing than following negative framing, and again lower following negative framing than following perfectly reliable predictions. That is, when unexpected or running against the prediction, a negative item would elicit the strongest amplification and therefore receive the lowest evaluation.

The opponent mechanism hypothesis, as a function of cognitive control, would imply counteractive response preparation (i.e., eye prepositioning toward the direction opposite to the prediction, and slowing of responses that match with the prediction). Opponent mechanisms on the basis of implicit processes (i.e., prediction error) should not affect eye prepositioning or response time.

## Results

### Experiment 1

In Experiment 1, in addition to the variations of cue types and food images, we manipulated the delay between the predictive cue and the target food image to examine whether the prior expectations strengthen over time^28,29^. If so, any influences from the predictive cues on the evaluative processing of the food images should be larger after long delays than after short delays.

For each subject, the data could be classified into 12 conditions by the type of outcome, the delay time and the type of predictive cue. There were two types of outcome, either positive or negative, and two levels of delay time, either 1 s or 9 s between predictive cue and target image. For the predictive cues, there were three types defined by the relationship between the predictive information and the actual outcome; a cue with 100% reliability was labeled as “Certain”; a cue with 50% reliability followed by the predicted outcome was labeled as “Valid 50%”; and a cue with 50% reliability followed by the opposite outcome was labeled as “Invalid 50%”.

#### Evaluation scores

**Figure 4:**
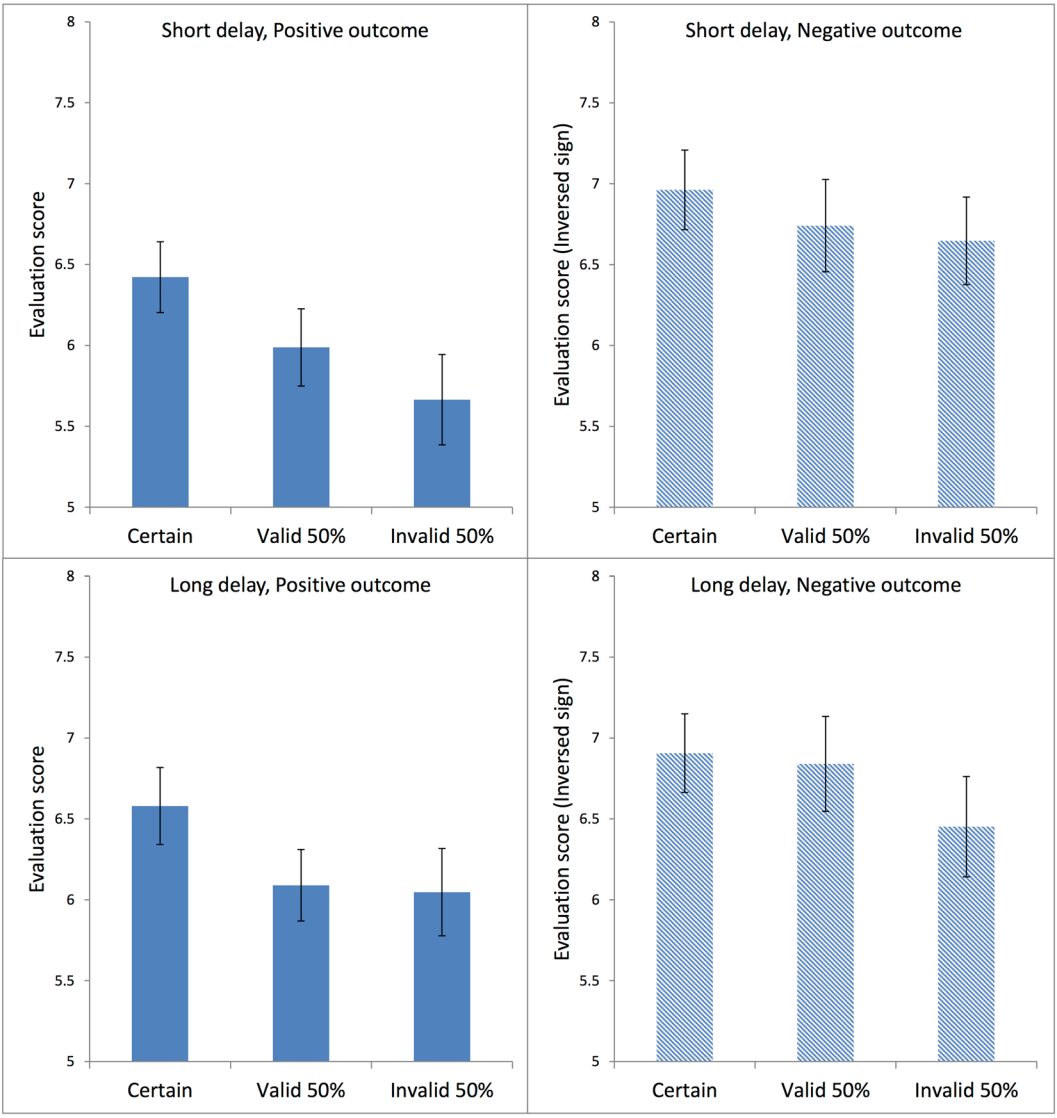
Average ratings in each condition in Experiment 1. Each panel shows data for Certain, Valid 50%, and Invalid 50% conditions. The top two panels are from the short-delay conditions; the bottom two panels from the long-delay conditions. The left two panels present the positive-outcome conditions; the right two panels, the negative-outcome conditions. The signs were inversed for the ratings in the negative outcome conditions in order to facilitate comparison with the positive outcome conditions. The error bars show the standard error of the mean in each condition.

In order to facilitate the comparison across conditions, for negative outcomes the sign of the given evaluation score was inversed. Figure 4 presents the average evaluation scores in each condition for Experiment 1. Regardless of outcome type and delay time, the evaluation scores appeared to be the highest for the Certain condition, and the lowest for the Invalid 50% condition. Negative outcomes appeared to be rated more extremely than positive outcomes. For statistical analysis of the average evaluation scores, we employed a three-factor analysis of variance (ANOVA) with repeated measures, in which the factors Outcome Type, Delay Time, and Cue Type were all within subjects.

The ANOVA produced a significant main effect of Cue Type, F(2,82) = 6.560, MSE = 1.716, η_p_^2^ = 0.138, p < 0.005, as well as a significant main effect of Outcome Type, F(1,41) = 4.906, MSE = 10.073, η_p_^2^ = 0.107, p < 0.05. There was no significant effect of Delay Time, F(1,41) = 1.068, MSE = 0.792, p = 0.307. Post-hoc pairwise comparisons using the Bonferroni test indicated that the average evaluation scores in the Certain condition were significantly higher than in the Valid 50% condition at p < 0.05, and also significantly higher than in the Invalid 50% condition at p < 0.05. The difference between the Valid 50% and the Invalid 50% condition was not significant, p = 0.274.

There was a significant interaction between Delay Time and Outcome Type, F(1,41) = 4.385, MSE = 0.503, η_p_^2^ = 0.097, p < 0.05. Post-hoc comparisons using the Bonferroni test showed, for short-delay trials, significantly more extreme evaluation scores for negative outcomes than for positive outcomes at p < 0.05, whereas in long-delay trials there was no significant difference between positive and negative outcomes, p = 0.653. There were no other significant interactions (all F values less than 2).

#### Manual response times

Response times were measured from the onset of the screen with the evaluation bar until a bend was detected in the joystick. By this definition, all trials in which the subject started bending the joystick before the onset of the screen with the evaluation bar were excluded from the analysis. For this reason, a total of 9.93% of all trials was rejected for the response time analysis. Two subjects started bending the joystick prematurely in every trial in some conditions; the data from these two subjects were excluded from the ANOVA analysis on response time.

**Figure 5:**
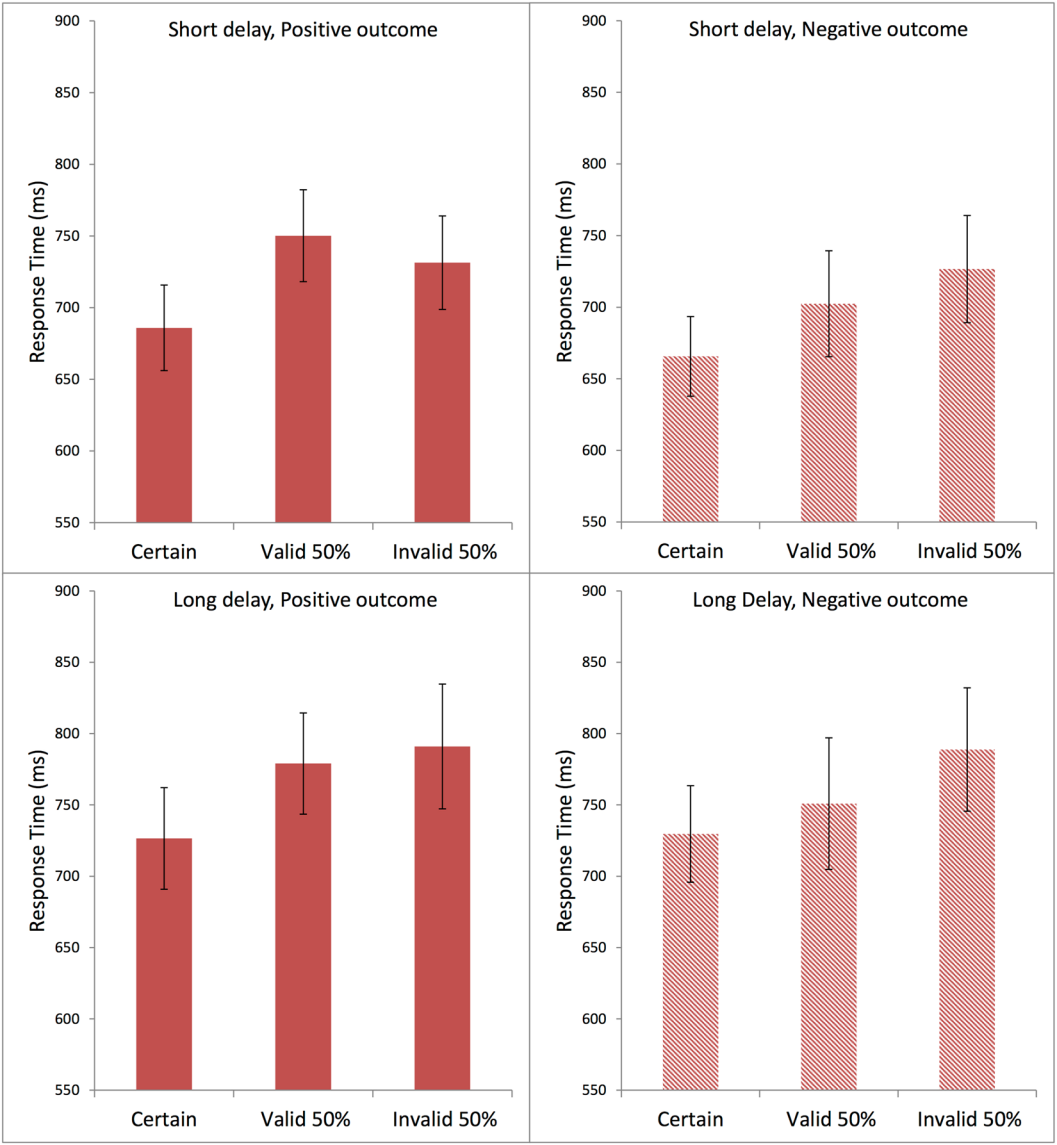
Average response times (ms) in each condition in Experiment 1. Each panel shows data for Certain, Valid 50%, and Invalid 50% conditions. The top two panels are from the short-delay conditions; the bottom two panels from the long-delay conditions. The left two panels present the positive-outcome conditions; the right two panels, the negative-outcome conditions. The error bars show the standard error of the mean in each condition.

Figure 5 presents the subjects’ average response times in Experiment 1, using the same format as for the evaluation scores in Fig. 4. Visual inspection suggested that the response times tended to be the fastest in the Certain condition and the slowest in the Invalid 50% condition.

Preliminary normality tests (Shapiro-Wilk) showed significant deviations from normality in the response times. For this reason, the subsequent statistical analysis of response times was conducted on a simple inverse transformation (1/RT)^49^, which yielded normal distributions. Statistical analysis, with a three-factor repeated measures ANOVA, completely within subjects, produced a significant main effect of Cue Type, F(2,78) = 9.163, MSE = 0.045, η_p_^2^ = 0.190, p < 0.001, and also of Delay Time, F(1,39) = 34.061, MSE = 0.039, η_p_^2^ = 0.466, p < 0.001. Outcome Type did not have a significant effect on the response time F(1,39) < 1. Post-hoc comparisons using the Bonferroni test indicated that the average response time in the Certain condition was significantly faster than in the Valid 50% condition at p < 0.01, and also significantly faster than in the Invalid 50% condition at p < 0.01. The difference between the Valid 50% and the Invalid 50% condition was not significant, p = 0.802.

There were no significant interactions (all F values less than 1.5).

#### Gaze distribution data

In order to examine whether the predictive cues elicited an anticipatory response bias, we analyzed the average gaze positioning during the delay period. A response bias should lead the subjects’ gaze positions to deviate toward the direction associated with the predicted outcome (in Experiment 1: negative – left, positive – right). This analysis of gaze positioning is focused on the blank screen period before target onset; at this time, before the outcome is known, the cue conditions can only be separated between 100% reliable and 50% reliable. In this analysis, then, the data were classified into eight conditions according to Delay Time (1 s or 9 s), Cue Valence (either positive or negative) and Cue Reliability (50% or 100%). For each subject in each condition, the average horizontal eye position was calculated during the delay relative to the blank screen.

**Figure 6:**
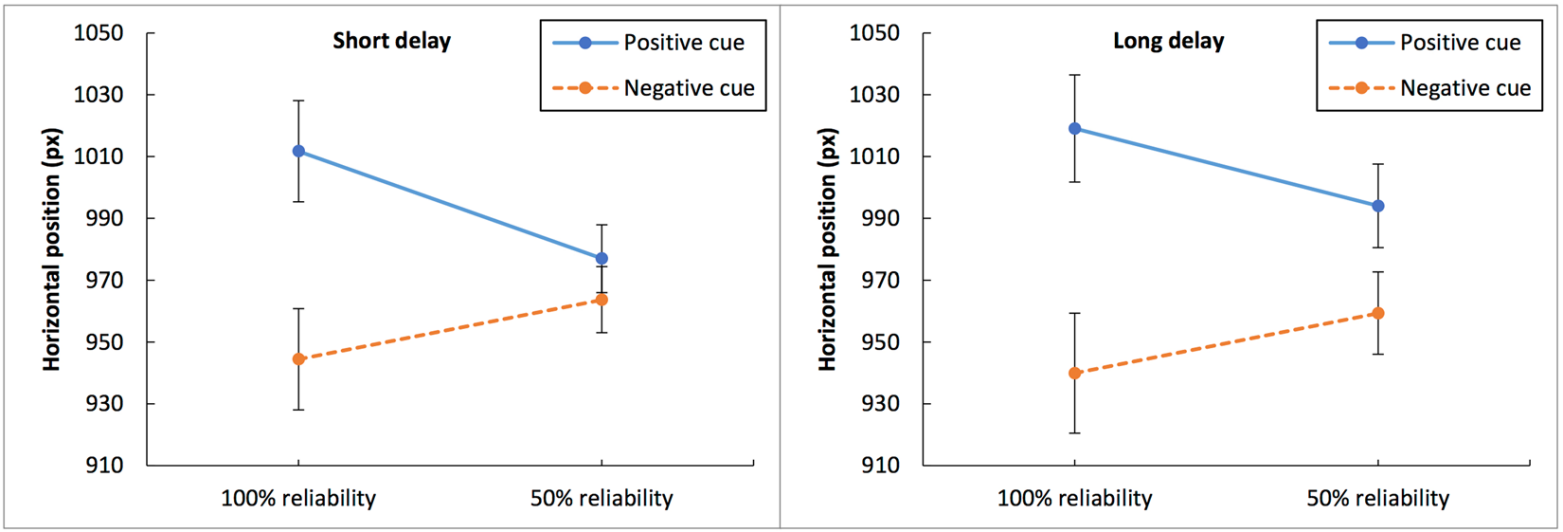
Average horizontal eye positions during the delay period in Experiment 1, as a function of delay duration, cue reliability, and cue valence. The Y-axis represents the horizontal position on the screen (the screen width is set to 1920 pixels). The left panel shows the data for the short-delay trials; the right panel, for the long-delay trials. The conditions with positive cues are shown in blue; with negative cues, in orange. The error bars represent the standard error of the mean.

Figure 6 presents the average horizontal eye positions during the delay as a function of condition in Experiment 1. For both delay durations, the average horizontal eye positions appeared to be shifted to the left or right depending on the cue valence, with more pronounced biases for 100% reliable cues and for longer delays.

A three-factor repeated measures ANOVA indicated that Cue Valence produced a statistically significant effect on the average horizontal eye positions, F(1, 41) = 6.937, MSE = 28559.298, η_p_^2^ = 0.145, p < 0.05. Delay Time, F(1, 41) < 1, and Cue Reliability, F(1, 41) = 3.297, MSE = 714.375, p = 0.077, did not produce a significant effect on the gaze positioning.

There was a significant interaction between Cue Valence and Delay Time, F(1, 41) = 6.402, MSE = 906.995, η_p_^2^ = 0.135, p < 0.05, and also between Cue Valence and Cue Reliability, F(1, 41) = 7.213, MSE = 7051.153, η_p_^2^ = 0.150, p = 0.010. There were no other interaction effects (the remaining F values < 1). Overall, the gaze positioning showed significant response biases associating positive cues with the rightward direction, negative cues with the leftward direction. These biases were particularly pronounced for long delays and 100% reliable cues.

### Experiment 2

In Experiment 2, we investigated further the effect of the reliability level of the predictive cues, as well as the response bias observed in the gaze positioning. One concern with the response bias was that in the previous experiment, we employed only one fixed scheme of mapping between spatial position and response (negative – left; positive – right). To ensure that the response bias followed from the prediction rather than any inherent spatial bias, we counterbalanced the mapping between spatial position and response across subjects in Experiment 2. Thus, we divided the subjects into two groups; one group was always presented with the default evaluation bar (as in Experiment 1; negative – left; positive – right); and the other group was always presented with a flipped evaluation bar (positive – left; negative – right).

With respect to cue reliability, instead of using certain versus random cues, in Experiment 2 we set a fixed reliability of 75% for both positive and negative cues, and further included a neutral cue type (a checkerboard, without positive or negative framing). Finally, in Experiment 2 the delay time was fixed at 2 s.

Thus, there were 12 conditions, with one between-subjects factor, Evaluation Bar (default versus flipped), and two within-subjects factors, Outcome Type (positive or negative) and Cue Type. For the Cue Type, there were three types defined by the relationship between the predictive information and the actual outcome; cues followed by the predicted outcome were labeled as “Valid 75%”; cues followed by an outcome opposite to the prediction were labeled as “Invalid 25%”; and cues that did not predict an outcome were labeled as “Neutral”.

#### Evaluation scores

**Figure 7:**
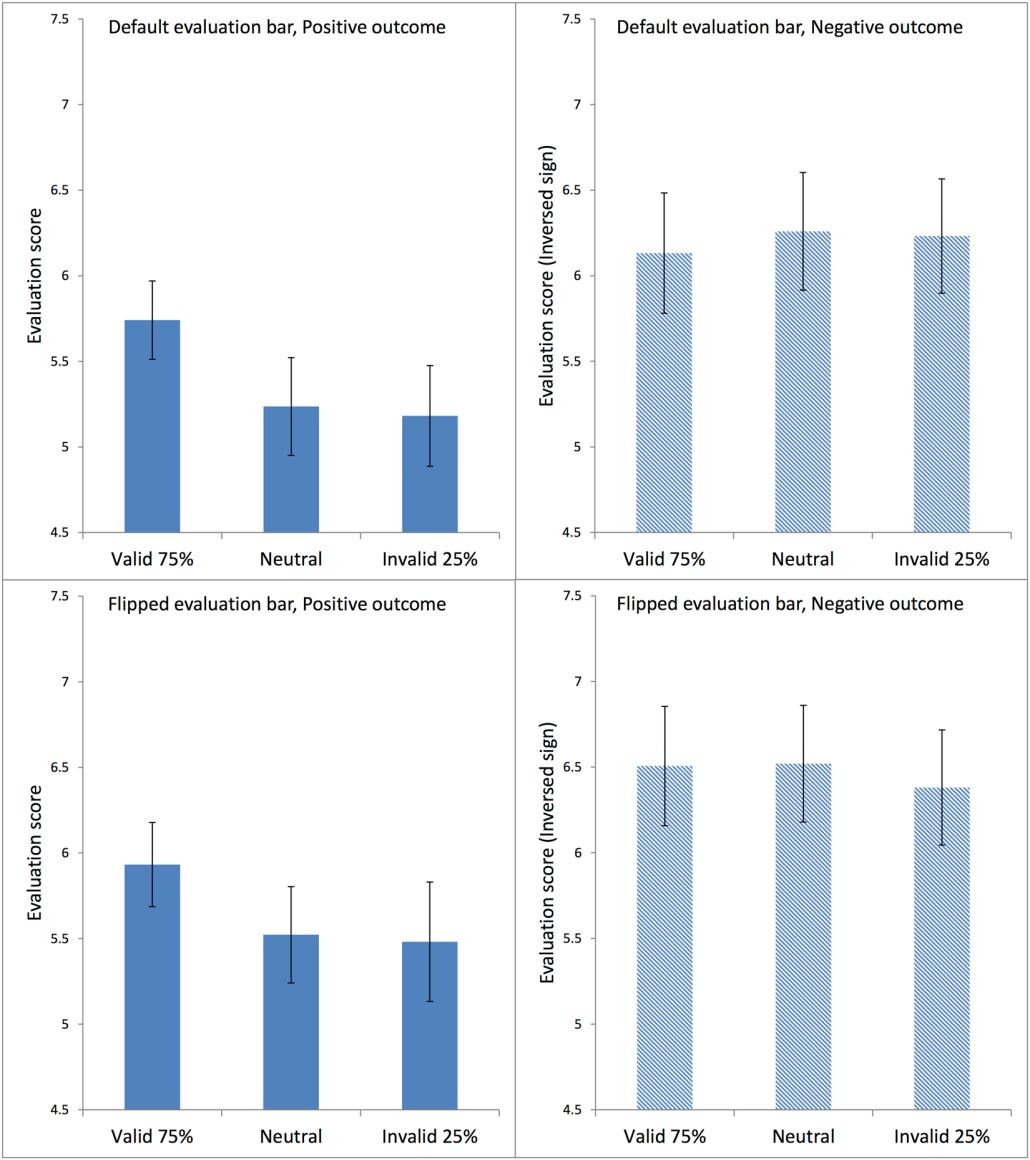
Average ratings in each condition in Experiment 2. Each panel shows data for Valid 75%, Neutral, and Invalid 25% conditions. The top two panels show data from participants who used the Default Evaluation Bar; the bottom two panels are from participants who used the Flipped Evaluation Bar. The left two panels present the positive-outcome conditions; the right two panels, the negative-outcome conditions. The signs were inversed for the ratings in the negative outcome conditions in order to facilitate comparison with the positive outcome conditions. The error bars show the standard error of the mean in each condition.

As before, for negative outcomes the sign of the given evaluation score was inversed. Figure 7 presents the average evaluation scores in each condition for Experiment 2. The predictions appeared to affect the evaluation scores for positive outcomes, but not for negative outcomes. For positive outcomes, the effects of the predictions again suggested spill-over rather than updating. For statistical analysis of the average evaluation scores, we employed a three-factor analysis of variance (ANOVA) with repeated measures, in which the factors Outcome Type and Cue Type were within subjects, whereas the factor Evaluation Bar was between subjects.

The ANOVA produced a statistically significant main effect of Outcome Type, F(1,64) = 10.071, MSE = 6.654, η_p_^2^ = 0.136, p < 0.005. The main effect of Cue Type was not statistically significant, F(2,128) = 2.934, MSE = 0.815, p = 0.057, nor was that of the between-subjects factor Evaluation Bar, F < 1.

There was a statistically significant interaction between Cue Type and Outcome Type in the average evaluation scores, F(2,128) = 7.120, MSE = 0.403, η_p_^2^ = 0.100, p < 0.005. Post-hoc pairwise comparisons, with the Bonferroni test, showed that for negative outcomes, there were no significant effects of cue type, whereas for positive outcomes, the average evaluation scores in the Valid 75% condition were significantly higher than those in the Invalid 25% condition at p < 0.05, and significantly higher than those in the Neutral condition at p < 0.001.

There were no two-way or three-way interactions with the between-subjects factor Evaluation Bar (all F values < 1).

#### Manual response times

**Figure 8:**
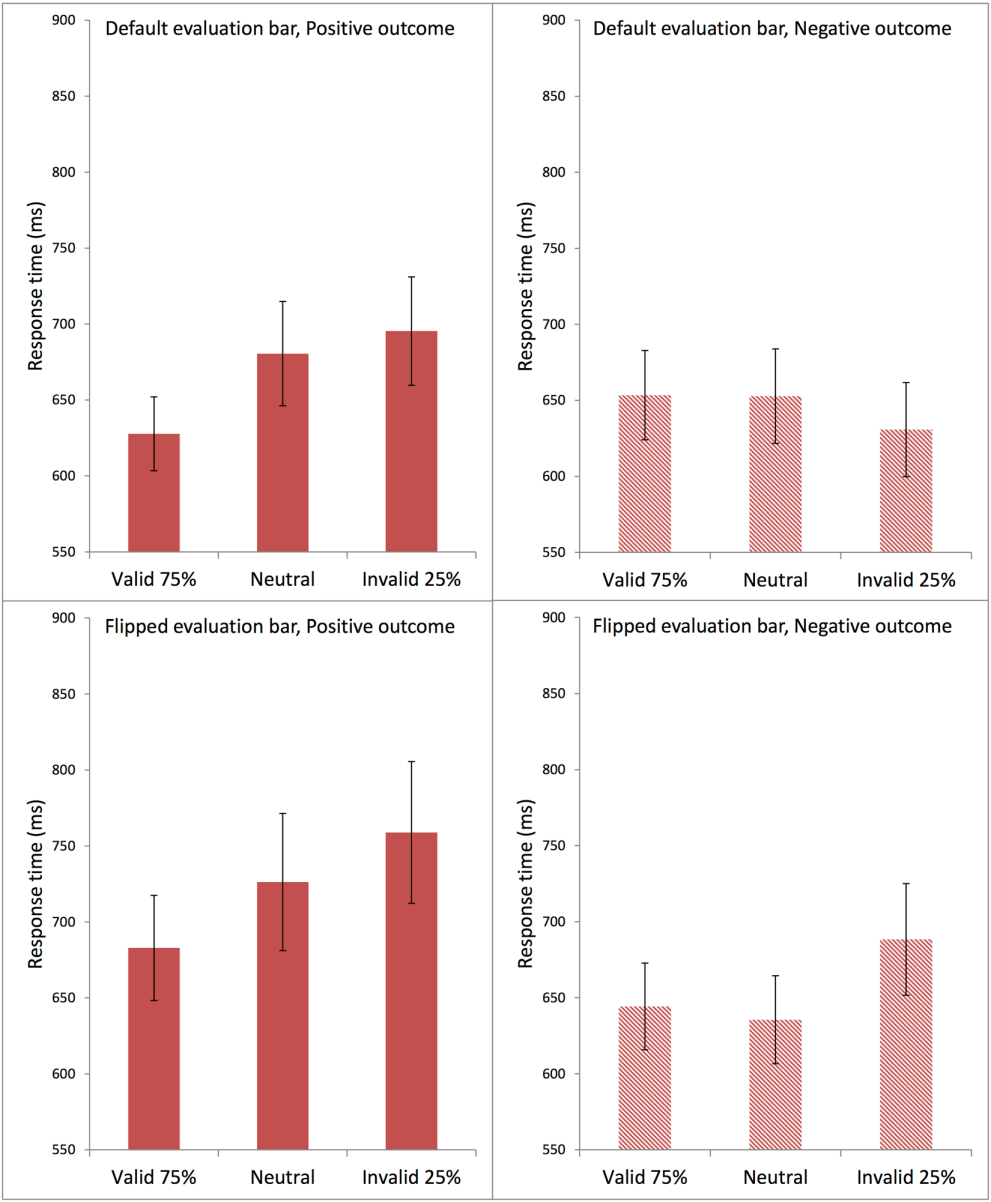
Average response times (ms) in each condition in Experiment 2. Each panel shows data for Valid 75%, Neutral, and Invalid 25% conditions. The top two panels show data from participants who used the Default Evaluation Bar; the bottom two panels are from participants who used the Flipped Evaluation Bar. The left two panels present the positive-outcome conditions; the right two panels, the negative-outcome conditions. The error bars show the standard error of the mean in each condition.

In order to prevent a loss of data due to premature bending of the joystick, in Experiment 2 the response method was modified by requiring the subjects to press the button on the joystick with their index finger for confirmation. Thus, response time was defined as the time between the onset of the response screen and the button press on the joystick. Figure 8 presents the average response times in each condition in Experiment 2, using the same format as Fig. 7. Overall the Valid 75% conditions appeared to produce the fastest response times, particularly for positive outcomes.

As in Experiment 1, preliminary normality tests (Shapiro-Wilk) showed significant deviations from normality in the response times; the subsequent statistical analysis was conducted on the inverse response times, which yielded normal distributions. A three-factor repeated measures ANOVA produced a significant main effect of Outcome Type, F(1,64) = 4.531, MSE = 0.118, η_p_^2^ = 0.066, p < 0.05. The factor Cue Type did not produce a significant main effect, F(2,128) = 2.605, MSE = 0.033, p = 0.078, nor did the between-subjects factor Evaluation Bar, F < 1. There was a statistically significant interaction between Cue Type and Outcome Type, F(2,128) = 3.905, MSE = 0.030, η_p_^2^ = 0.058, p < 0.05. Post-hoc pairwise comparisons using the Bonferroni test showed that, for positive outcomes, there was a significant difference between the Valid 75% condition and the Invalid 25% condition at p < 0.01, but not between the Valid 75% and the Neutral conditions, p = 0.056, nor between the Neutral and the Invalid 25% conditions, p = 0.530. Conversely, for negative outcomes, there were no significant differences as a function of Cue Type (all p values above 0.7).

There was no significant interaction between Cue Type and the between-subjects factor Evaluation Bar, F(2,128) = 2.771, MSE = 0.033, p = 0.066, nor between Outcome Type and Evaluation Bar, F < 1. The three-way interaction was also not significant, F(2,128) = 1.796, MSE = 0.030, p = 0.170.

#### Gaze distribution data

As in Experiment 1, we analyzed the gaze positioning during the delay period in Experiment 2 to examine whether the predictive cues elicited a response bias. In this analysis, focused on the blank screen period before target onset (i.e., when the outcome is not yet known), the data were classified into six conditions by the between-subjects factor Evaluation Bar (default or flipped) and the within-subjects factor Cue Type (either positive, neutral, or negative). For each subject in each condition, the average horizontal eye position was calculated during the delay relative to the blank screen.

**Figure 9:**
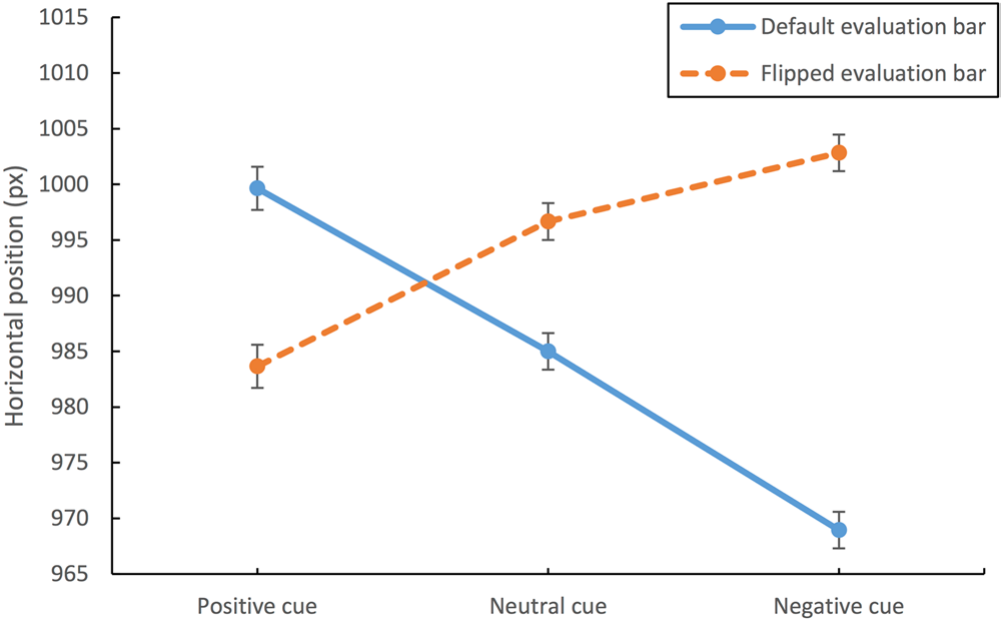
Average horizontal eye positions during the delay period in Experiment 2, as a function of the type of cue and evaluation bar. The Y-axis represents the horizontal position on the screen (the screen width is set to 1920 pixels). The data with the Default Evaluation Bar are shown in blue; with the Flipped Evaluation Bar, in orange. The error bars represent the standard error of the mean.

Figure 9 presents the average horizontal eye positions during the delay as a function of condition in Experiment 2. The data appeared symmetrical for the two groups of subjects, suggesting that the gaze positioning flipped as a function of evaluation bar alignment, implementing response biases (toward the negative or positive pole depending on the prediction).

In the subsequent ANOVA, in order to facilitate the comparison with different evaluation bar alignments, we used the inverse of the horizontal eye position data in the flipped evaluation bar condition; that is, we subtracted the given horizontal eye positions from the maximal horizontal pixel value (1920). This procedure aligns the horizontal eye position data of the two groups in the same direction, so that any response biases yield similar numerical values across the evaluation bar conditions.

A repeated measures ANOVA confirmed that the within-subjects factor Cue Type had a statistically significant impact on the gaze positioning during the blank screen period, F(2,128) = 40.023, MSE = 257.261, η_p_^2^ = 0.385, p < 0.001. Post-hoc pairwise comparisons using the Bonferroni test showed that there were statistically significant differences in the average horizontal eye positions at p < 0.001 for all pairwise comparisons among the three cue types. The horizontal eye positions leaned most toward the positive pole following positive cues (M = 967.992, SD = 7.872), and most toward the negative pole following negative cues (M = 943.061, SD = 6.667), implying more central positioning after neutral cues (M = 954.169, SD = 6.726).

Also the between-subjects factor Evaluation Bar produced a significant effect on the average horizontal eye positions, F(1,64) = 18.088, MSE = 9495.071, η_p_^2^ = 0.220, p < 0.001. However, there was no interaction between Cue Type and Evaluation Bar, F(2,128) = 2.493, MSE = 257.261, p = 0.087.

## Discussion

Two experiments were conducted to examine the effects of predictive cues on the evaluation of single food images. The experiments pitched the congruent bias hypothesis, as a function of framing^23–29^ or more implicit processes (e.g., priming or emotional contagion)^30–37^, against the opponent mechanism hypothesis, derived from the concepts of proactive control^38–42^ and prediction error^45–48^. In Experiment 1, we found that the evaluation scores were influenced by the predictive cues, such that 100% reliable cues produced more extreme scores than 50% reliable cues, both for aversive and for appetitive food images, suggesting the operation of a congruent bias. The response times showed slower responses following 50% reliable cues than following 100% reliable cues, further implicating a selective response preparation in line with the predictions. Gaze prepositioning during the delay period, after prediction but before the food image, also suggested a response bias in accordance with the predictions.

Experiment 2 provided corroborating evidence, again showing that the evaluation scores were influenced by the predictive cues, with 75% reliable cues leading to more extreme scores for correct predictions than for incorrect predictions, suggesting congruent bias effects, particularly in the case of appetitive food images. Once again, the response times showed slower responses following incorrect predictions than following correct predictions, indicating that prior expectation guided the selective response preparation. Gaze prepositioning during the delay period also exhibited a response bias in accordance with the predictions, regardless of the spatial alignment of positive versus negative poles.

Positive predictive cues elicited an anticipatory process that set up a congruent response bias, leading to gaze prepositioning in the spatial direction associated with the positive pole of the evaluation bar. This response bias caused appetitive images to be evaluated more positively while (in case of erroneous prediction) causing aversive images to be evaluated less negatively. Conversely, the opposite processes occurred for negative predictions. Crucially, these anticipatory processes and response biases reflected active inferences^3,4^, depending on the perceived reliability of the predictive cues, with stronger effects for more reliable cues.

Taken together, both experiments provided solid evidence in favor of the congruent bias hypothesis. As a corollary, the data charted how external influences from prior (“objective”) information can have a significant impact on subsequent (“subjective”) evaluative processing, underscoring the potential importance of this line of research for other domains of value-based decision-making (e.g., in politics and bioethics).

### Congruent Bias as an Active Process toward Confirmation

Several elements in the data suggested a role for active, deliberative processing in response to the predictive information rather than merely effects from automatic associations. The effects of cue reliability on evaluation scores suggested voluntary control of expectation. In Experiment 1, the congruent bias was large following cues with 100% reliability, but there was no significant difference between the 50% Valid and 50% Invalid condition. In Experiment 2, the 75% reliable cues did have an impact on the evaluation scores, particularly for positive outcomes (with more extreme scores in the 75% Valid condition than in the 25% Invalid condition). The pattern of data suggested that the subjects set their expectations based on the perceived reliability of the predictive cues – strongly for highly reliable cues, somewhat for reasonably reliable cues, and not so much for unreliable cues. Most likely, this was due to actively controlled usage of the predictive cues.

Here, it should be noted that the active processing of predictive information does not necessarily preclude the existence of concurrent, more implicit types of interference. A useful approach in this regard, compatible with the concept of framing^23–29^, may be that of the somatic marker hypothesis^50–52^. Applying this hypothesis to the current data, we might suggest that the covert action of “marker” signals underpinned the introduction of a congruent bias in the selection of an aversive or appetitive mode of processing. Importantly, this hypothesis contends that it is erroneous to limit human decision-making to mechanisms of either conditioning alone or cognition alone. Thus, while the concurrent operation of conditioning cannot – should not – be excluded, the relevant point here is that the cue reliability determined the degree of impact from predictive information. This finding implicates cognitive control and active processing.

Apart from the dependence of evaluative processing on the reliability of the predictions, two further aspects of the data suggest that the impact of external influences was mediated via active, cognitive control. Notably, the gaze prepositioning indicated a selective preparation aligned with the expected direction of response. This gaze prepositioning was likewise sensitive to the cue reliability, with more pronounced prepositioning for more reliable predictive cues. Also, the response times indicated that the evaluative processing was more effortful following incorrect predictions than following correct predictions, again suggesting an active form of inference relative to the earlier prediction. Once more, the effects on response times depended on the reliability of the predictive cues.

The effortful, slower evaluative processing following incorrect predictions produced less extreme scores than the faster evaluative processing following correct predictions. For instance, an appetitive food image presented after a positive cue tended to quickly receive a highly positive evaluation, whereas an appetitive food image presented after a negative cue tended to receive a less positive evaluation after a longer deliberation, as if the subject tried to accommodate the prediction (finding something negative in the image, or trying to attenuate the discrepancy between prediction and outcome by reducing the evaluation). This may be thought of as an active attempt to assimilate a surprising stimulus to a prior expectation^53^. Interestingly, there appeared to be an asymmetry, particularly in Experiment 2, with 75% reliable cues showing influences from prediction on the evaluation of appetitive images, but not on the evaluation of aversive images. This suggests that, relatively speaking, the evaluative processing of appetitive images is more vulnerable to external influences. Negative predictions tend to distort the reception of appetitive images, whereas aversive images appear to be more immune to positive framing.

In conclusion, the predictions produced a congruent response bias that can best be characterized as an active confirmation bias. Depending on the perceived reliability, positive predictive cues elicited an active positive anticipation of the food images, whereas negative cues elicited an active – and relatively stronger – negative anticipation. Correct predictions produced quick and amplified evaluation scores. Erroneous predictions produced slow and less extreme evaluation scores in an apparent effort to accommodate the prediction.

## Methods

### Subjects

In Experiment 1, there were 42 subjects. All were Kyushu University students (26 males, 16 females) with a mean age of 22.45 ± 3.63 years. In Experiment 2, there were 66 subjects. All were Kyushu University students (38 males, 28 females) with a mean age of 23.94 ± 4.54 years. There were 3 left-handed subjects in Experiment 1, and 2 in Experiment 2; however, these subjects also used their right hand to manipulate the joystick.

In both experiments, all subjects had normal or corrected-to-normal vision. Each person was given 1000 yen as a compensation for the participation, which lasted less than 1 hour. All subjects gave informed consent, and reported that they were in healthy condition before and after the experiment.

### Apparatus and stimuli

The visual stimuli were presented in a dimly lit room on a 23.8-inch full high definition flat-panel-monitor, with a display resolution of 1920 × 1080 pixels. The subjects were seated approximately 62 cm from the monitor. To minimize head movement a chin-rest with a forehead-support was used. The evaluation responses were recorded using a joystick (Logitech, Switzerland; model no. 963290-0403). Eye positions were recorded using Eye Tribe, an eye-tracking device at 60 Hz sampling rate (The Eye Tribe Aps, Denmark); a system with sufficient reliability for present purposes^22,54,55^.

In order to start the eye tracking, the subject was asked to follow a dot on the screen for a 16-point calibration. After the calibration, the gaze coordinates were calculated through Eye Tribe with an average error of less than 0.5^◦^ visual angle on the 23.8-inch display. To prevent heat buildup a small universal serial bus (USB) fan was used. All events and recordings were controlled through code written in Psychopy (version 1.84.2)^56,57^ including the PyTribe library.

All visual stimuli were presented as inset images on a white background in the middle of the otherwise black screen. The size of the inset image was fixed at 380 × 380 pixels for the predictive cues, and at 600 × 600 pixels for the food images. The predictive cues were icons: a tray for positive; a hazard sign for negative; and a checkerboard for neutral. Different colors were used (counterbalanced across subjects) to indicate the reliability of the cues in Experiment 1. Food images were drawn from the FoodCast research image database (FRIDa)^58^ and supplemented with non-copyrighted images to construct a set of 200 food images with a balanced range of appetitive and aversive stimuli. If necessary, images were resized to fit in the frame of 600 × 600 pixels. The images were classified into 100 appetitive and 100 aversive stimuli based on the categorization by FRIDa and ratings by lab members. The categorizations proved valid for all 200 images based on the average responses of all subjects in Experiments 1 and 2: Each stimulus in the category of aversive food images received on average a negative rating, whereas each stimulus in the category of appetitive food images received on average a positive rating.

### Experimental procedures

#### Experiment 1

Participants were asked to evaluate 180 naturalistic food images in 3 consecutive blocks of 60 trials with breaks of not more than 5 minutes between the blocks. At the start of each trial, the word “short” or “long” was presented for 1 s in the middle of the screen to indicate the delay time between the predictive cue and the target image, either 1 s or 9 s. Next the predictive cue was shown at the center of the screen for 1 s, followed by the blank screen for the delay period. Then the target image was shown for 2 s, and in turn replaced by the response screen. The subject had maximally 6 s to evaluate the food image by bending the joystick to move the cursor on the evaluation bar from −10 to 10. The bending angle was used to indicate the evaluation score. After the response was made, there was a blank screen for 2 s as inter-trial interval (ITI).

Different icons were used for the predictive cues to indicate the outcome, either appetitive or aversive, whereas the color indicated the reliability level of the cue, either 100% or 50% reliability. The color assignment was counterbalanced across subjects. The reliability of the predictive cue was further indicated numerically in percentage, presented in small print beneath the icon.

The subjects were instructed to give their evaluation for each food image, focusing on the appeal of the image, not their general preference of the pictured food item. The evaluations had to be given on a continuous rating scale from −10 to 10, with a value of −10 for a maximally disgusting food image, a value of +10 for a maximally attractive food image, and a value of 0 for a food image that was neither likeable nor disgusting. The subjects were instructed to start bending the joystick for evaluation only after the evaluation bar appeared on the screen.

It was explained to the subjects that the cues predicted whether the upcoming food image would be likeable or dislikeable, with different levels of certainty, either 100% certain or 50%.

Before the experimental session, the subjects were given the opportunity to practice controlling the joystick for up to 30 times. The experiment included 180 trials, consisting of 15 repetitions of each of the 12 conditions, with 3 levels Cue Type (Certain, Valid, Invalid), 2 levels of Outcome Type (Positive, Negative), and 2 levels of Delay Time (1 s, 9 s). No food image was presented more than once. The 180 trials were presented in pseudorandom order to ensure that each block of 60 trials contained 5 repetitions of each condition.

#### Experiment 2

Participants were asked to evaluate 200 naturalistic food images in 4 consecutive blocks of 50 trials with breaks of not more than 5 minutes between the blocks. The procedures were the same as in Experiment 1 except for the following. The delay time between the predictive cue and the food image was fixed at 2 s; and no word cue was given to indicate the delay time at the beginning of the trial. The cue reliability for the positive and negative cues was fixed at 75%, and a third type of cue was included (a neutral cue, represented by a checkerboard as icon).

The evaluation bar assignment was changed for 2 groups of subjects, with either a conventional alignment (negative – left; positive – right) or the opposite alignment. Here, subjects were asked to confirm their evaluation by clicking the trigger on the joystick.

The experiment included 200 trials, divided into 6 conditions, with 3 levels Cue Type (Valid 75%, Neutral, Invalid 25%) and 2 levels of Outcome Type (Positive, Negative). The Valid 75% conditions consisted of 60 repetitions, whereas the Invalid 25% and the Neutral conditions each consisted of 20 repetitions. No food image was presented more than once. The 200 trials were presented in pseudorandom order to ensure that each block of 50 trials contained the same distribution of trials per condition.

### Ethics statement

The protocols for the present study were designed in accordance with the Declaration of Helsinki, and were approved by the Human Ethics Committee of the Faculty of Arts and Science, Kyushu University (issue number 201711). Informed consent was obtained in writing from each subject.

## Electronic supplementary material

Supplementary Information
